# Impact of acid selection on thrombelastometric test results - an in-vitro cohort study

**DOI:** 10.1186/s12959-026-00912-2

**Published:** 2026-08-20

**Authors:** Gerrit U. Herpertz, Johannes Altmann, Bernhard H. Rauch, Christian F. Weber, Simon T. Schäfer, Greta Bischof-Westermann

**Affiliations:** 1 Department of Anaesthesiology, Intensive Care Medicine, Emergency Medicine and Pain Medicine, Carl von Ossietzky Universität Oldenburg and Klinikum Oldenburg, Rahel-Straus-Straße 10, 26133 Oldenburg, Germany; 2 Division of Pharmacology and Toxicology, University Medicine Oldenburg, Carl von Ossietzky Universität Oldenburg, Oldenburg, Germany; 3 Department of Anaesthesiology, Intensive Care, Emergency Medicine and Pain Therapy, Schön Klinik Hamburg Eilbek, Hamburg, Germany; 4 Department of Anaesthesiology, Intensive Care Medicine and Pain Therapy, University Hospital Frankfurt, Goethe University Frankfurt, Frankfurt, Germany

## Abstract

**Background:**

Acidosis is common in critically ill patients and is associated with coagulopathy. The impact of different acids on coagulation remains poorly understood. Hydrochloric acid has often been used without critical evaluation. We aimed to investigate the influence of different acids and their concentrations on rotational thromboelastometry (ROTEM).

**Methods:**

In this observational study ten, blood samples were treated with hydrochloric acid (HCl), lactic acid (C₃H₆O₃), phosphoric acid (H₃PO₄), or dihydrogen phosphate (H₂PO₄⁻) to achieve defined pH levels (7.2–6.9). Respiratory acidosis was simulated by incubation in a 20% CO₂ atmosphere. Five additional samples were exposed to varying acid concentrations. Samples were analyzed using the ROTEM EXTEM assay. Clotting time (CT), clot formation time (CFT), α-angle, maximum clot firmness (MCF), and maximum lysis (ML) were assessed.

**Results:**

All acids exhibited concentration-dependent effects on EXTEM parameters. HCl caused significant reductions in MCF and prolonged CFT, indicating impaired clot firmness and propagation. C_3_H_6_O_3_ induced moderate changes, with elevated fibrinolysis at intermediate concentrations. H₃PO₄ reduced MCF and increased ML, while H₂PO₄⁻ primarily delayed clotting initiation and markedly inhibited fibrinolysis. CO₂-induced acidosis (pH ≈ 7.1) led to reduced fibrinolytic activity without affecting CT or MCF. Across acid types, increasing acidity consistently impaired clotting dynamics.

**Conclusion:**

The type of acid inducing acidosis was associated with distinct alterations in ROTEM parameters in vitro. The choice of acid should be carefully considered in experimental models and taken into account when interpreting data. Whether such effects occur in vivo remains uncertain.

**Supplementary Information:**

The online version contains supplementary material available at 10.1186/s12959-026-00912-2.

## Introduction

The body’s acid-base equilibrium, specifically a pH maintained within the range of approximately 7.35 to 7.45, is vital for many enzymatic reactions and physiological processes, including blood coagulation [1]. Acidosis is a pathophysiologic condition characterized by an excess acid load in the body’s fluids, often resulting in reduced blood pH. This pathologic state can be categorized as either metabolic or respiratory acidosis, with the former caused by an increase in acid due to metabolic processes or insufficient renal acid excretion, and the latter due to elevated carbon dioxide levels in the blood, usually as a result of inadequate respiration [1]. Alterations in blood pH play a critical role in haemostasis. Acidosis is documented to affect various stages of haemostasis, including platelet adhesion, aggregation, coagulation cascade, and fibrinolysis [2–4]. Specifically, acidemia exerts direct inhibitory effects on platelet function and coagulation factor activity, leading to diminished thrombin generation and impaired clot stability [2]. In sum, acidosis can significantly influence blood properties and dynamics including coagulation mechanisms.

To address the problem of acidosis and its effects on blood coagulation, various approaches have been described in the literature for measuring the influence of pH changes. However, there has been no systematic clarification of the potentially different effects depending on the type of acid used to adjust the pH value. It is possible to test various acids and, alternatively, to expose the blood to CO₂. Hendriks et al. have highlighted the challenges of precisely adjusting blood pH due to the complexity of its buffering systems [5]. Because of this, many studies have ultimately opted to add a fixed amount of concentrated hydrochloric acid (HCl; 0.05 to 5 M), followed by measurement of the resulting pH, accepting a certain degree of variability in the results, e.g. aiming for 6.8 resulting in pH-levels between 6.75 and 6.87 [6–9]. 

Even if hydrochloric acid has been used for adjusting the pH value in several ex-vivo experiments, it does not naturally occur in the body nor the bloodstream, apart from its presence in the stomach [10]. In contrast, several other acids (e.g. H₂PO₄⁻) play a more physiologically relevant role, for example as part of buffering systems [11]. These systems consist of a weak acid and its corresponding base, allowing them to stabilize pH fluctuations within a certain range specific to the buffer system. Examples include the bicarbonate and phosphate buffer systems.

From a physiological perspective, acidosis can be induced by controlled CO₂ addition. The bicarbonate buffer is an open system influenced by respiration: hypoventilation increases CO₂, shifting the equilibrium to the right and increasing hydronium ion release [11]. The phosphate buffer system is particularly important in intra- and extracellular environments. Phosphate is a component of the inorganic structure of the bone and it is excreted as H₂PO₄⁻ via the kidneys to eliminate acids [11]. The protein buffer system based on proteins has numerous acidic and basic groups, which can bind to or release hydrogen ions. Haemoglobin in red blood cells is a significant protein buffer; it binds to excess hydronium ions to form HHb (acid-haemoglobin), reducing the concentration of H^+^ in the blood, and thus raising the blood pH [12]. Another biologically relevant acid is lactic acid (C_3_H_6_O_3_), which plays a crucial role in anaerobic metabolism [1]. 

Furthermore, an important aspect to consider is the effect of adding highly concentrated acid to a blood sample. Before dilution occurs, haemolysis and/or protein denaturation are likely to take place. Likewise, if the concentration is too low, dilution will occur until the target pH is reached, which also negatively influences coagulation [13]. Although a relevant impact can be assumed it has not been systematically studied so far. Furthermore, using different acids leads to different ion concentrations, which may result in specific complex formation reactions with plasma constituents and direct interactions with the active sites of coagulation proteins. In vitro, certain anions can chelate calcium or form complexes with clotting factors, thereby altering their conformation and enzymatic activity [14, 15]. 

ROTEM is a viscoelastic testing method that provides rapid, point-of-care assessment of clot formation and stability and is widely used to guide haemostatic therapy in settings with complex coagulopathy, such as trauma, cardiac surgery, and major haemorrhage. Technological advances have improved automation, usability, and speed, expanding its clinical applications across diverse patient populations. However, ROTEM has important limitations, including limited sensitivity to therapeutic anticoagulants and certain congenital bleeding disorders, as well as technical constraints related to in vitro testing and restricted pediatric approval [16]. 

This study aimed to systematically evaluate the impact of pH titration with different acids and concentrations on clot initiation, propagation, and lysis as assessed by the ROTEM EXTEM assay.

## Materials and methods

### Participants

In summary, fifteen participants were recruited. Inclusion criteria were age ≥ 18 years and exclusion criteria were intake of anticoagulatory medication within the last two weeks and hereditary coagulopathy. No formal a priori power calculation was performed, as this study was designed as an exploratory mechanistic investigation.

### Sample collection

Blood samples were obtained from a cubital vein by venipuncture into a 3 mL tube containing 0.3 mL 0.106 M sodium-citrate (Sarstedt, Nümbrecht, Germany). Samples were processed within four hours.

### Sample preparation and pH adjustment

pH was measured in 750 µL blood with the pH meter SD20 (Mettler Toledo, Columbus, USA) using the InLab Semi-Micro pH electrode (Mettler Toledo, Columbus, USA) at 37 °C (Matrix orbital delta plus; IKA-Werke GmbH & Co. KG, Staufen, Germany). The temperature was controlled by the puncture thermometer Labtherm (Dostmann electronic GmbH, Wertheim, Germany). The pH meter was calibrated each day with calibration solutions of pH 4.006, 6.865 and 9.180 (NIST/DIN buffer; Mettler Toledo) at 37 °C. The blood from all tubes of each participant was pooled in a 50 mL tube (Sarstedt, Nümbrecht, Germany) to obtain a homogeneous sample.

Different pH values: The blood samples of ten participant, 15 mL each, were adjusted to pH 7.2, 7.1, 7.0 and 6.9 with HCl (0.5 M), C_3_H_6_O_3_ (0.5 M), H₃PO₄ (0.5 M) and H₂PO₄⁻ (1 M). In addition, 3 mL of blood of each participant was incubated in a cell culture flask (T-25; Sarstedt, Nümbrecht, Germany) at an atmosphere of 20% CO₂ (CO2 incubator ICO 105, Memmert GmbH & Co. KG, Schwabach, Germany) for 60 min. For every examination an untreated control sample was analysed.

Different acid concentrations: The blood samples of five participant, 9 mL each, were adjusted to pH 7.0 with HCl (VWR International GmbH, Darmstadt, Germany), C_3_H_6_O_3_ (VWR International GmbH, Darmstadt, Germany), H₃PO₄ (Sigma-Aldrich, Taufkirchen, Germany) and NaH₂PO₄ (neoFroxx GmbH, Einhausen, Germany) at different concentrations (1.0 M, 0.5 M, 100 mM, ). Concentration ranges were informed by previously published studies; however, reported low-concentration approaches could not be reliably reproduced under our experimental conditions. Therefore, we focused on concentration levels that allowed for consistent and measurable pH shifts within the experimental system.

A pH tolerance of ± 0.015 was accepted during the titration process for all targeted pH values. To exclude any hemolysis effects, all sampels were inspected during and after processing. No macroscopic evidence of haemolysis was observed.

### ROTEM analysis

Two ROTEM Delta devices (3000 and 4000-series; Werfen GmbH, Munich, Germany) were used for the thrombelastometric analysis. The self-validated EXTEM analysis (data not shown) was performed for all blood samples as described by the manufacturer. Quality control was performed as recommended by the manufacturer.

### Statistics

Results are presented as means and standard deviations. Data distribution was assessed using the Shapiro–Wilk test. Depending on the distribution, comparisons between groups were performed using Student’s t-test or Mann–Whitney U test for two groups, and analysis of variance (ANOVA) or Kruskal–Wallis test for multiple groups, with strict Bonferroni correction applied for multiple comparisons as appropriate. We used SPSS (IBM, Version 29.0) for statistical calculations. Significance level was defined as *p* = 0.05 and adjusted internally according to Bonferroni correction where appropriate.

## Results

**Fig. 1:**
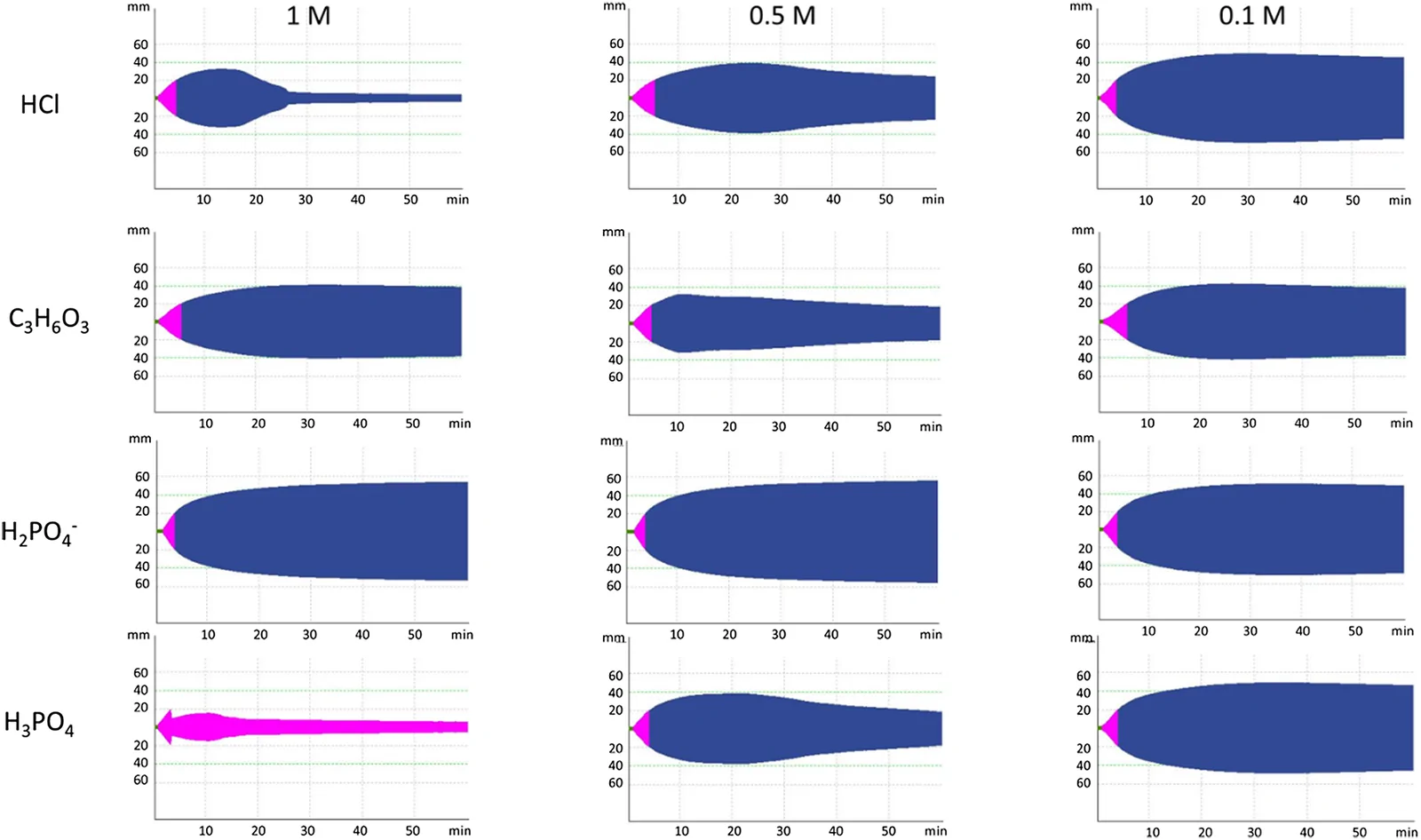
Exemplary TEMograms, comparison of citrate whole blood samples acidified to pH 7.0 by titration with four acids each at 3 stock concentrations

**Fig. 2:**
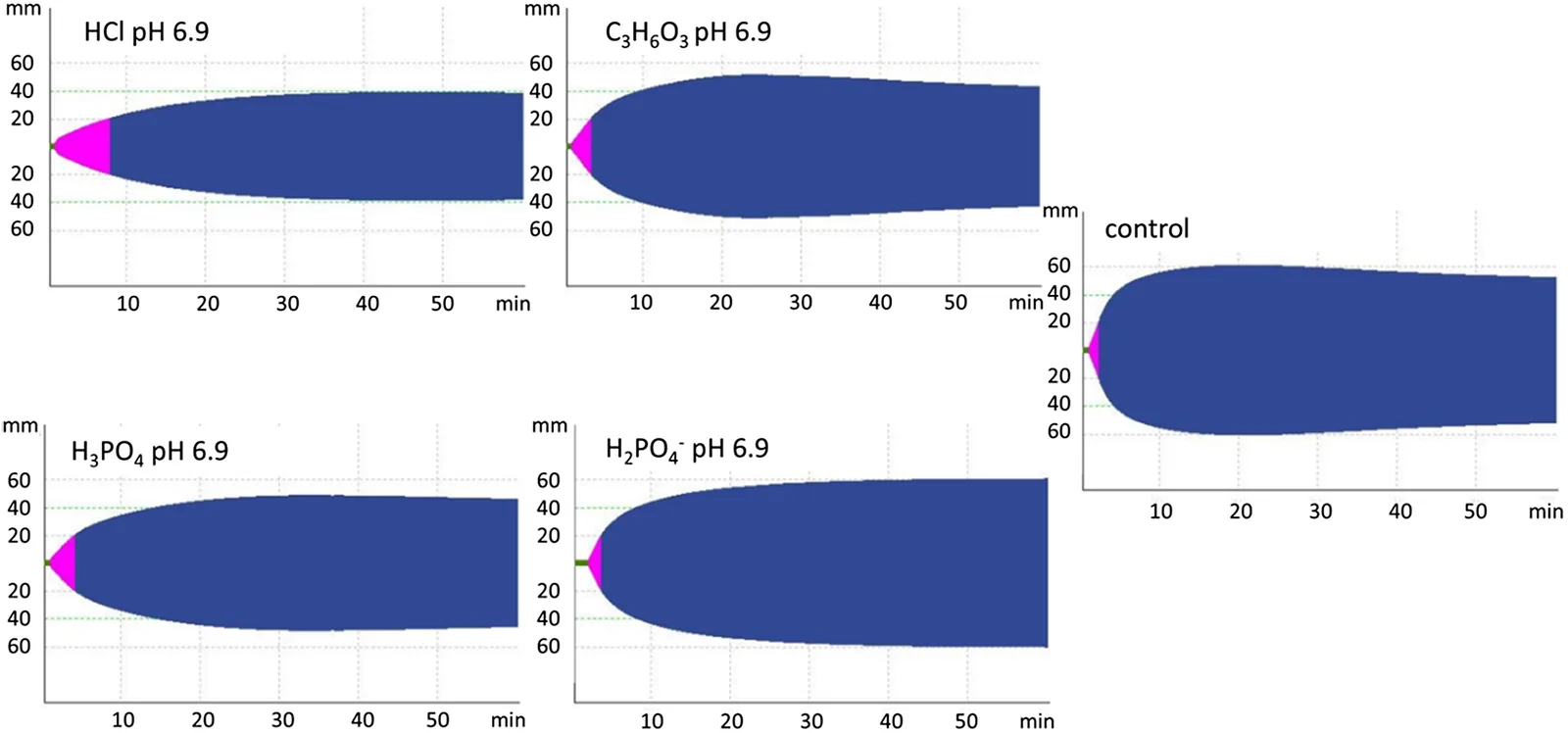
Representative EXTEM TEMograms, comparison between a control citrate whole blood sample and those acidified to pH 6.9 by titration with 4 acids each at a stock concentration of 0.5 M

The samples were collected and processed between January 2025 and February 2025 at the University Department of Anaesthesiology, Intensive Care, Emergency Medicine and Pain Therapy of the Carl von Ossietzky University Oldenburg. The reported ROTEM parameters only refer to the EXTEM assay. We recruited five participants (four male, one female; mean age 37.2 ± 14.2 years) for the concentration experiment at one acidic pH (Table 1), and ten participants (six male, four female; mean age 39.9 ± 15.2 years) for the pH comparison study (Table 2). Exemplary TEMograms for Table 1 data are presented in Fig. 1 and for Table 2 data in Fig. 2. The results for the different acids and concentrations are displayed in Table 1 and exemplary TEMograms are shown in Fig. 1. The volumes required to achieve the respective pH values are listed in the supplementary Table 1.

**Table 1:** Comparison of the effect of different acid stock concentrations used to achieve pH 7.0 on ROTEM EXTEM parameters. EXTEM parameters are shown for the used acids and the concentrations of 1 M, 0.5 M and 0.1 M to achieve a pH of 7.0

Acid	CT [s]	CFT [s]	MCF [mm]	α-angle [°]	ML [%]	CFR [°]
HCl 1 M	45.7 ± 1.2	300.7 ± 71.6	34.6 ± 9.8	65.7 ± 11.5	49.0 ± 42.9	71.0 ± 6.3
HCl 0.5 M	51.0 ± 5.2	236.0 ± 119.5	43.3 ± 8.4	56.3 ± 14.7	20.7 ± 16.6	64.0 ± 9.5
HCl 0.1 M	48.0 ± 2.7	179.0 ± 82.3	52.0 ± 10.8	60.3 ± 10.2	5.7 ± 3.2	66.3 ± 6.5
p-value	0.241	0.346	0.069	0.666	0.210	0.549
C_3_H_6_O_3_ 1 M	46.0 ± 10.5	188.3 ± 95.3	48.0 ± 11.4	64.3 ± 15.0	7.0 ± 2.7	69.0 ± 11.0
C_3_H_6_O_3_ 0.5 M	53.7 ± 10.2	171.0 ± 78.5	43.0 ± 10.2	62.0 ± 12.1	41.3 ± 35.1	66.0 ± 9.9
C_3_H_6_O_3_ 0.1 M	52.3 ± 9.0	182.3 ± 97.6	50.7 ± 12.5	57.3 ± 14.6	10.0 ± 1.7	62.7 ± 12.6
p-value	0.624	0.972	0.717	0.828	0.152	0.794
H₃PO₄ 1 M	46.0 ± 4.6	171.5 ± 137.9	35.0 ± 14.0	65.7 ± 11.2	51.6 ± 42.4	72.7 ± 5.5
H₃PO₄ 0.5 M	50.3 ± 3.2	160.3 ± 57.7	48.7 ± 11.6	64.3 ± 9.5	19.7 ± 28.9	67.7 ± 7.2
H₃PO₄ 0.1 M	63.3 ± 14.6	141.33 ± 42.5	54.3 ± 8.5	63.0 ± 6.6	4.3 ± 3.8	66.0 ± 6.0
p-value	0.125	0.906	0.191	0.941	0.218	0.451
H₂PO₄⁻ 1 M	72.3 ± 17.0	103.7 ± 42.6	59.7 ± 8.3	70.7 ± 7.6	0	72.7 ± 6.7
H₂PO₄⁻ 0.5 M	81.7 ± 25.8	96.3 ± 42.3	62.0 ± 7.9	71.7 ± 7.6	0	73.0 ± 7.2
H₂PO₄⁻ 0.1 M	55.3 ± 8.3	130.0 ± 56.4	55.7 ± 9.9	65.3 ± 10.1	5.7 ± 0.6	67.3 ± 8.4
p-value	0.284	0.678	0.686	0.643	< 0.001	0.606

Data are presented as mean +/- SD (*N* = 5); CT: Clotting Time, CFT: Clot Formation Time, MCF: Maximum Clot Formation, ML: Maximum Lysis, CFR: Clot Formation Rate

### Comparison of effect of acid stock concentration on ROTEM parameters at pH 7

#### Hydrochloric acid

CT was lowest at 1 M (45.7 ± 1.2 s), slightly higher at 0.1 M (48.0 ± 2.7 s), and reached its maximum at 0.5 M (51.0 ± 5.2 s; *p* = 0.241). CFT shortened steadily with decreasing concentration, from 300.7 ± 71.6 s (1 M) to 179.0 ± 82.3 s (0.1 M; *p* = 0.346). MCF rose progressively with dilution, from 34.6 ± 9.8 mm at 1 M to 52.0 ± 10.8 mm at 0.1 M (*p* = 0.069), with a significant difference between 1 M and 0.1 M (*p* = 0.010). The α-angle varied inconsistently (1 M = 65.7 ± 11.5°, 0.5 M = 56.3 ± 14.7°, 0.1 M = 60.3 ± 10.2°; *p* = 0.666). ML declined sharply with decreasing acid strength, from 49.0 ± 42.9% (1 M) to 5.7 ± 3.2% (0.1 M; *p* = 0.210), whereas CFR remained stable (71.0 ± 6.3 to 66.3 ± 6.5 mm/min; *p* = 0.549). Relative to the control (CT 50.0 ± 4.4 s, CFT 87.6 ± 17.75 s, MCF 57.9 ± 10.8 mm, ML 17.9 ± 9.2%, CFR 74.6 ± 2.95 mm/min), 1 M HCl produced faster clot initiation but weaker clot firmness and increased fibrinolysis. At 0.5 M, CT and CFR approximated control values, though CFT was still prolonged. The 0.1 M solution showed near-control parameters with distinctly lower ML.

#### Lactic acid

CT values ranged from 46.0 ± 10.5 s (1 M) to 53.7 ± 10.2 s (0.5 M) and 52.3 ± 9.0 s (0.1 M; *p* = 0.624). CFT remained comparable across concentrations (188.3 ± 95.3 s to 182.3 ± 97.6 s; *p* = 0.972). MCF showed only minor variation (48.0 ± 11.4 mm at 1 M, 43.0 ± 10.2 mm at 0.5 M, 50.7 ± 12.5 mm at 0.1 M; *p* = 0.717). The α-angle (*p* = 0.828) showed no clear trend. ML peaked at 0.5 M (41.3 ± 35.1%) and was markedly lower at 1 M (7.0 ± 2.7%) and 0.1 M (10.0 ± 1.7%; *p* = 0.152). CFR remained stable (*p* = 0.794).

Compared to control, C_3_H_6_O_3_ caused only minor deviations: CT and CFT were similar, MCF was slightly reduced, and the α-angle modestly decreased. ML was substantially higher at 0.5 M, while other concentrations were near control levels.

#### Phosphoric acid

CT increased gradually from 46.0 ± 4.6 s (1 M) to 63.3 ± 14.6 s (0.1 M; *p* = 0.125). CFT varied widely without a consistent trend (*p* = 0.906). MCF rose with dilution, from 35.0 ± 14.0 mm (1 M) to 54.3 ± 8.5 mm (0.1 M; *p* = 0.191). The α-angle remained largely unchanged (*p* = 0.941). ML declined markedly as concentration decreased—51.6 ± 42.4% (1 M) to 4.3 ± 3.8% (0.1 M; *p* = 0.218)—while CFR was stable (*p* = 0.451).

Relative to the control, 1 M H₃PO₄ led to lower MCF (35.0 mm) and enhanced fibrinolysis (ML 51.6%). At 0.1 M, CT was prolonged but other parameters approximated control, except for the lower ML.

#### Dihydrogen phosphate

CT was prolonged at higher concentrations (72.3 ± 17.0 s at 1 M, 81.7 ± 25.8 s at 0.5 M, 55.3 ± 8.3 s at 0.1 M; *p* = 0.284), whereas CFT (*p* = 0.678), MCF (*p* = 0.686), α-angle (*p* = 0.643), and CFR (*p* = 0.606) remained stable. ML was completely suppressed at 1 M and 0.5 M (0%), but measurable at 0.1 M (5.7 ± 0.6%; *p* < 0.001).

Compared to control, H₂PO₄⁻ primarily inhibited fibrinolysis at higher concentrations (ML = 0%, *p* < 0.001) with only moderate CT prolongation and no substantial changes in other parameters.

##### pH dependence of ROTEM parameters by titrating acid

Compared to baseline conditions, acidification significantly altered coagulation parameters in a pH- and acid-dependent manner. HCl caused the most pronounced impairment, with a marked prolongation of CFT (up to ~ 430 s at pH 6.9) and a progressive decrease in MCF (to 37.6 mm) and α-angle (to 59.1°). Clot firmness and kinetics deteriorated consistently with decreasing pH, accompanied by reduced ML and CFR at lower pH levels. C₃H₆O₃ showed moderate effects. CFT was significantly prolonged and MCF slightly reduced, particularly at lower pH. The α-angle and CFR declined mainly at pH 6.9, while ML showed mild reductions. H₃PO₄ demonstrated a similar but slightly less pronounced pattern than lactic acid, with moderately prolonged CFT and reduced MCF at lower pH. ML decreased significantly across all pH levels, and CFR declined at pH 6.9. In contrast, H₂PO₄⁻ exhibited a different profile. CFT remained close to or below native values at higher pH and increased only at pH 6.9. MCF and α-angle were largely preserved, while ML showed a marked and consistent reduction across all pH levels, approaching complete suppression at pH 6.9. Results are displayed at Table 2.

At all investigated pH levels, coagulation parameters differed significantly between acids, with consistent patterns across conditions. Results are shown in Table 3 and TEMogram examples in Fig. 2. At pH 7.2, HCl showed the most impaired clot formation, with markedly prolonged CFT and reduced MCF compared to all other acids. In contrast, H₂PO₄⁻ exhibited the shortest CFT and highest MCF and α-angle. Lactic acid and H₃PO₄ showed intermediate profiles, with moderately prolonged CFT and slightly reduced clot firmness. At pH 7.1, these differences persisted. HCl remained associated with significantly prolonged CFT and reduced MCF compared to all other acids, whereas H₂PO₄⁻ maintained the highest MCF and α-angle. Lactic acid and H₃PO₄ again showed comparable intermediate effects. At pH 7.0, intergroup differences became more pronounced. H₂PO₄⁻ demonstrated significantly higher CT, MCF, and α-angle compared to all other acids, along with the lowest ML. HCl continued to show the most impaired clot propagation (highest CFT, lowest MCF), while lactic acid and H₃PO₄ remained intermediate. At pH 6.9, HCl exhibited the most severe coagulopathy, with extreme prolongation of CFT and the lowest MCF and α-angle. H₂PO₄⁻, in contrast, preserved clot firmness and kinetics (highest MCF and α-angle, lowest ML), despite a marked prolongation of CT. Lactic acid and H₃PO₄ again showed moderate impairment, with similar values between both acids. Across all pH levels, ML was consistently lowest under H₂PO₄⁻ conditions, whereas CFR and α-angle generally paralleled MCF.

### Respiratory acidosis model

After one hour of incubation with CO₂, the pH decreased from 7.467 ± 0.05 to 7.106 ± 0.07 (*p* < 0.001). ML and CFR were significantly lower (*p* = 0.007, *p* = 0.031) compared to the native samples. No significant differences were observed in CT, CFT, MCF or α-angle. Results are shown in Table 2.

**Table 2:** Comparison of ROTEM EXTEM parameters between native samples and acidified samples at 4 different pHs. Here the different ROTEM parameters are shown for every acid used to achieve pH levels 7.2, 7.1, 7.0 and 6.9. They were compared to the unprocessed native results

	pH	CT [s]	CFT [s]	MCF [mm]	α-angle [°]	ML [%]	CFR [mm/min]
nat		52.3 ± 4.6	87.6 ± 17.8	56.1 ± 4.4	72.7 ± 3.6	13.4 ± 3.2	74.6 ± 3.0
CO₂		53.6 ± 4.5	100.9 ± 20.3	58.4 ± 4.1	70.1 ± 4.0	8.5 ± 3.1**	72.0 ± 2.9**
HCl	7.2	46.1 ± 4.6**	197.5 ± 84.5***	45.2 ± 5.4***	64.4 ± 10.8	14.8 ± 13.2	69.5 ± 5.8*
	7.1	48.1 ± 6.1	227.1 ± 68.5***	44.5 ± 5.0***	67.1 ± 8.1	7.3 ± 6.5**	71.2 ± 5.0
	7.0	47.8 ± 5.2	257.7 ± 115.2***	42.7 ± 4.4***	64.0 ± 11.1*	9.1 ± 9.9	69.7 ± 5.5*
	6.9	47.4 ± 3.4*	429.5 ± 174.8***	37.6 ± 5.4***	59.1 ± 10.6***	3.6 ± 6.5**	67.2 ± 4.3***
C_3_H_6_O_3_	7.2	43.6 ± 4.9***	131.3 ± 27.9***	50.1 ± 4.2**	66.0 ± 5.7**	11.4 ± 3.0	70.1 ± 4.2*
	7.1	47.4 ± 8.1	120.2 ± 30.0**	53.6 ± 4.4	68.7 ± 5.2	9.7 ± 3.2*	71.3 ± 3.4*
	7.0	44.5 ± 3.5***	123.1 ± 32.2**	53.6 ± 4.5	68.2 ± 5.2*	8.7 ± 2.4**	70.7 ± 3.5*
	6.9	49.6 ± 6.6	174.2 ± 59.4***	48.2 ± 5.3**	60.6 ± 8.1***	9.3 ± 3.8*	64.4 ± 6.4***
H_3_PO_4_	7.2	40.9 ± 6.4***	129.4 ± 30.00**	50.3 ± 4.1**	68.4 ± 6.1	9.6 ± 4.0*	70.7 ± 4.6*
	7.1	43.8 ± 3.4***	129.6 ± 38.1**	52.5 ± 4.5	68.7 ± 6.5	7.7 ± 2.2**	71.2 ± 4.6
	7.0	43.2 ± 5.4**	129.9 ± 32.1**	52.7 ± 3.9	69.5 ± 6.1	6.4 ± 3.7***	71.7 ± 4.2
	6.9	46.7 ± 4.2*	184.3 ± 57.0***	48.6 ± 4.6**	61.6 ± 9.4**	4.7 ± 2.4***	66.1 ± 6.2**
H_2_PO_4_^−^	7.2	45.6 ± 5.7*	115.7 ± 34.6	53.1 ± 4.6	70.8 ± 5.9	6.6 ± 2.5***	72.5 ± 4.5
	7.1	51.2 ± 8.1	101.0 ± 26.8	57.8 ± 4.1	72.6 ± 4.5	4.2 ± 2.4***	73.5 ± 4.4
	7.0	60.1 ± 11.7	94.8 ± 23.4	58.8 ± 3.7	72.8 ± 3.8	1.9 ± 1.8***	74.0 ± 3.8
	6.9	91 ± 23.4**	113.3 ± 32.9*	57.0 ± 4.8	69.8 ± 4.4	0.2 ± 0.6***	71.4 ± 4.7

Data are presented as mean +/- SD (*N* = 10). Parameter comparisons are between native blood and acidfied samples. * *p* < 0.05, ** *p* < 0.01, *** *p* < 0.001

## Discussion

In our experiments, acidosis induced by different acids and concentrations caused distinct, acid-dependent changes in ROTEM EXTEM parameters. HCl, lactic acid, and H₃PO₄ all reduced MCF, with HCl showing the strongest effect, CT was accelerated at pH ≤ 7.2 for all three acids compared to control. In contrast, H₂PO₄⁻ prolonged CT only at more severe acidosis (pH 6.9) and markedly suppressed fibrinolysis. In the respiratory acidosis model (CO₂ incubation), pH dropped to ~ 7.1, reducing ML and CFR while CT, CFT, MCF, and α-angle remained unchanged.

**Table 3:** Comparison of the effect of different acids on ROTEM EXTEM parameters

Acid	CT [s]	CFT [s]	MCF [mm]	α-angle [°]	ML [%]	CFR [°]
**pH 7.2**						
HCl ^a^	46.1 ± 4.6	197.5 ± 84.5	45.2 ± 5.4	64.4 ± 10.8	14.8 ± 13.2	69.5 ± 5.8
C_3_H_6_O_3_^b^	43.6 ± 4.9	131.3 ± 27.9	50.1 ± 4.2	66.0 ± 5.7	11.4 ± 3.0	70.1 ± 4.2
H_3_PO_4_^c^	40.9 ± 6.4	129.4 ± 30.0	50.3 ± 4.1	68.4 ± 6.1	9.6 ± 4.0	70.7 ± 4.6
H_2_PO_4_^− d^	45.6 ± 5.7	53.1 ± 4.6	53.1 ± 4.6	70.8 ± 5.9	6.6 ± 2.5	72.5 ± 4.5
post hoc test *p* < 0.05	%	a-b, a-c, a-d	a-c	%	%	%
**pH 7.1**						
HCl ^a^	48.1 ± 6.1	227.1 ± 68.5	44.5 ± 5.0	67.1 ± 8.1	7.3 ± 6.5	71.2 ± 5.0
C_3_H_6_O_3_^b^	47.4 ± 8.1	120.2 ± 30.0	53.6 ± 4.4	68.7 ± 5.2	9.7 ± 3.2	71.3 ± 3.4
H_3_PO_4_^c^	43.8 ± 3.4	129.6 ± 38.1	52.5 ± 4.5	68.7 ± 6.5	7.7 ± 2.2	71.2 ± 4.6
H_2_PO_4_^− d^	51.2 ± 8.1	101.0 ± 26.8	57.8 ± 4.1	72.6 ± 4.5	4.2 ± 2.4	73.5 ± 4.4
post hoc test *p* < 0.05	%	a-b, a-c, a-d	a-b, a-c, a-d	%	%	%
**pH 7.0**						
HCl ^a^	47.8 ± 5.2	257.7 ± 115.2	42.7 ± 4.4	64.0 ± 11.1	9.1 ± 9.9	69.7 ± 5.5
C_3_H_6_O_3_^b^	44.5 ± 3.5	123.1 ± 32.2	53.6 ± 4.5	68.2 ± 5.2	8.7 ± 2.4	70.7 ± 3.5
H_3_PO_4_^c^	43.2 ± 5.4	129.9 ± 32.1	52.7 ± 3.9	69.5 ± 6.1	6.4 ± 3.7	71.7 ± 4.2
H_2_PO_4_^− d^	60.1 ± 11.7	94.8 ± 23.4	58.8 ± 3.7	72.8 ± 3.8	1.9 ± 1.8	74.0 ± 3.8
post hoc test *p* < 0.05	a-d, b-d, c-d	a-b, a-c, a-d	a-b, a-c, a-d, b-d, c-d	%	b-d, c-d	%
**pH 6.9**						
HCl ^a^	47.4 ± 3.4	429.5 ± 174.8	37.6 ± 5.4	59.1 ± 10.6	3.6 ± 6.5	67.2 ± 4.3
C_3_H_6_O_3_^b^	49.6 ± 6.6	174.2 ± 59.4	48.2 ± 5.3	60.6 ± 8.1	9.3 ± 3.8	64.4 ± 6.4
H_3_PO_4_^c^	46.7 ± 4.2	184.3 ± 57.0	48.6 ± 4.6	61.6 ± 9.4	4.71 ± 2.4	66.1 ± 6.2
H_2_PO_4_^− d^	91 ± 23.4	113.3 ± 32.9	57.0 ± 4.8	69.8 ± 4.4	0.2 ± 0.6	71.4 ± 4.7
post hoc test *p* < 0.05	b-d, c-d	a-b, a-c, a-d, c-d	a-b, a-c, a-d, b-d, c-d	b-d	b-c, b-d, c-d	b-d

Data are presented as mean ± SD (*N* = 10). Acidification was achieved using 0.5 M acid in all cases. Significant differences at each pH between any 2 acids are indicated (*p* < 0.05)

### General effect of acidosis on ROTEM

Progressive acidosis, particularly at pH values below 7.1, markedly impairs the enzymatic activity of critical coagulation complexes such as tissue factor–VIIa and factor Xa–Va. According to Meng et al., enzymatic activity is reduced by up to 90% as pH approaches 7.0 [17]. The increasing concentration of hydrogen ions disrupts the ionic interactions necessary for the assembly and function of coagulation complexes on negatively charged phospholipid surfaces of activated platelets. As a result, even when plasma concentrations of coagulation factors in vivo are within normal range, their functionality may be markedly compromised in the acidotic environment typical of major trauma or massive transfusion. This highlights the critical importance of early recognition and correction of acidosis in coagulopathic bleeding [18]. Although plasma coagulation as assessed by CT was not impaired by hydronium ions alone, ROTEM does not account for endothelial contributions to haemostasis. This may partially explain why pronounced in vitro ROTEM alterations do not necessarily translate directly into clinically apparent coagulopathy in vivo.

### Influence of concentration

#### Haemolysis and protein denaturation

Highly concentrated acids initially create a strongly acidic microenvironment at the drop-in before they are fully diluted and buffered by the surrounding medium. This transient, localized acidosis may lead to protein denaturation and haemolysis, both of which are critical concerns in vitro [19]. 

Moreover, coagulation factors, as proteins with complex tertiary structures, are particularly sensitive to pH fluctuations. In a highly acidic milieu, their function could potentially be impaired through structural changes or degradation, which may lead to altered thrombin generation, impaired fibrin polymerization, or disruption of the balance between procoagulant and anticoagulant pathways [20]. 

#### HCl

Several studies have employed HCl at varying concentrations to induce acidosis. Some added a fixed volume to the blood, which represents a major methodological difference from our model [6, 8, 9]. Such an approach may induce extended haemolysis and proteolysis, particularly at higher acid concentrations [19, 20]. Engström et al. observed a prolongation of CFT and a reduction in α-angle, findings comparable to our results [8]. Dirkmann et al. applied different concentrations to achieve distinct pH levels and examined the combined impact on ROTEM parameters together with hypothermia. The closest temperature to body temperature was 36 °C [9]. While the overall trend of their results aligns with ours, our observed effects were more pronounced and taken at 37° C. In a separate investigation, Dirkmann et al. reported that severe acidosis (pH 6.9) produced a clot lysis index of 80%, whereas moderate acidosis (pH 7.1) resulted in 95% [21]. In contrast, our findings indicated that ML remained unaffected at both pH levels. Other ROTEM parameters were not reported in this study.

#### Lactic acid (C_3_H_6_O_3_)

Gissel et al. employed 3 M C_3_H_6_O_3_ to achieve a pH of 7.0. They reported markedly prolonged CT (> 400 s), while MCF remained unchanged, and the α-angle was increased in the acidic sample [22]. Activation was performed using their own tissue factor rather than the commercial activator reagent. The MCF findings are consistent with our results, and we similarly observed an increase in α-angle with rising C_3_H_6_O_3_ concentrations. Shenkman et al. used 13.3 mM C_3_H_6_O_3_ to target pH 7.0–7.1 in diluted blood within a trauma-induced coagulopathy model. They applied tissue factor at a 1:50 dilution relative to the standard reagent, rendering CT results incomparable to ours. Despite this difference, MCF and α-angle were unaffected by C_3_H_6_O_3_ [23]. 

#### Phosphoric acid (H_3_PO_4_) and Dihydrogen phosphate (H_2_PO_4_^-^)

To the best of our knowledge, there are no studies about the use of H_3_PO_4_ and H_2_PO_4_^−^ in this context. In two studies, phosphate-buffered saline (PBS, pH 5.4), containing H₂PO₄⁻ and HPO₄²⁻, was used. Ramaker et al. applied 1 mL 50mmol/L PBS pH 5.4 to induce acidosis assessing coagulation with the TEG500 system. They reported changes in all variables: r-time (comparable to CT) and k-time (comparable to CFT) were prolonged, whereas α-angle and maximal amplitude decreased [24]. The CT and CFT findings align with our observations for H₂PO₄⁻, while α-angle and MCF results more closely correspond to H₃PO₄. Lv et al. also used PBS (pH 5.8) with the TEG500 system, targeting pH 6.95, 7.15, and 7.35. They observed prolongation of r-time and k-time, whereas α-angle and maximal amplitude remained unchanged [25]. These findings are consistent with our results for H₂PO₄⁻.

### Influence of the acid

#### Effect of acid concentration

At a certain value there are differences in dilution, ion concentration and calcium complexation depending on the acid used to modulate pH. Due to the small sample size, the results of this experiment are underpowered and only indicate a trend. HCl at 1 M markedly delayed clot propagation, as evidenced by prolonged CFT, accompanied by reduced MCF. These effects diminished at lower concentrations. C_3_H_6_O_3_ produced milder alterations, with increased ML observed predominantly at 0.5 M, suggesting enhanced clot breakdown under moderate acidity, while other parameters remained largely unaffected across concentrations. H₃PO₄ significantly impaired clot firmness at higher concentrations and increased fibrinolysis, indicating weaker clots prone to degradation. Interestingly, CT was prolonged at lower concentrations, suggesting complex interactions with coagulation factors. H₂PO₄⁻ primarily suppressed fibrinolysis at 0.5 M and 1 M, with minimal effect on other parameters, indicating a potent antifibrinolytic action relevant in acidotic conditions.

The acids differ sharply in dissociation behavior at room temperature, pKa values given in Table 4. HCl (pKₐ ≈ -8) ionizes completely and immediately increases proton concentration. C_3_H_6_O_3_ (pKₐ = 3.86), although weak, is almost fully dissociated at physiological pH and shows little change upon titration to lower values. H₃PO₄ (pKₐ₁ = 2.12) dissociates mostly to H₂PO₄⁻, which gradually shifts to H₂PO₄⁻/HPO₄²⁻ equilibrium when titrating toward pH 6.9. Using NaH₂PO₄ instead of H₃PO₄ raises total phosphate concentration. The differences at 37 °C are insignificant [26–28]. 

**Table 4:** pKa values of the used acids at room temperature [28, 29]

Acid	HCl	C_3_H_6_O_3_	H_3_PO_4_	H_2_PO_4_^−^
pKa	-8	3.86	2.12	7.21

#### Fibrinpolymerisation

A study by Kurniawa et al. showed that phosphate ions strongly inhibit the lateral aggregation of fibrin protofibrils without affecting fibrinogen cleavage or protofibril formation. As a result, fibrin networks formed in the presence of phosphate-containing buffers consist of thinner, less branched fibers with reduced permeability, while their mechanical stiffness (elastic modulus) remains largely unchanged. This effect is independent of the specific buffer chemistry and also occurs in plasma. The authors suggest that physiological fluctuations in phosphate or bicarbonate levels may modulate clot architecture in vivo, regulating permeability without compromising mechanical integrity [4]. 

Chloride ions have a significant modulatory effect on fibrin polymerization by specifically inhibiting the lateral aggregation of protofibrils. This leads to the formation of thinner and more curved fibrin fibers compared to those formed in the presence of inert anions like fluoride. The effect is pH-dependent and becomes more pronounced at alkaline pH values (above 7.4), where chloride likely interacts with a basic residue in fibrin with a pKₐ around 9.2. As the pH decreases toward more acidic values (e.g., pH 7.0 or lower), this inhibitory effect diminishes due to protonation of the target residue, resulting in thicker and straighter fibrin fibers. Thus, chloride ions might act as physiological regulators of fibrin architecture, with their influence being reduced under mildly acidic conditions such as those found in inflamed or ischemic tissues. Interestingly, the addition of HCl introduces both a high concentration of chloride ions and a drop in pH. Although the elevated chloride level would theoretically enhance the inhibitory effect on fibrin structure, the concurrent acidification may override this effect by suppressing chloride’s binding through protonation of the fibrin target site. Consequently, dense and thick fibrin fibers form despite the presence of excess chloride [29]. 

#### Clotting time

The shortened CT observed under acidic conditions is unlikely to reflect an actual enhancement of enzymatic coagulation activity. Instead, it may result from altered physicochemical interactions within the assay system or differential impairment of endogenous anticoagulant pathways, leading to an apparent acceleration of clot initiation [22]. 

The addition of H₃PO₄ to a blood sample has a markedly different effect compared to adding H₂PO₄⁻, primarily due to differences in dissociation behavior and pH impact. H₃PO₄ is a triprotic acid that dissociates stepwise, and at physiological pH, it rapidly donates protons, leading to a significant drop in pH and shifting the equilibrium toward H₂PO₄⁻ and monohydrogen phosphate [30]. In contrast, adding H₂PO₄⁻ directly increases the phosphate ion concentration within the existing physiological buffer range, without disrupting the pH balance. This explains the difference in CFT comparing H₃PO₄ and H₂PO₄⁻.

The isolated prolongation of CT in the presence of H₂PO₄⁻ with MCF remaining unaffected, indicates that phosphate primarily delays the initiation phase of coagulation rather than impairing fibrin polymerization or clot stability. This might be explained by mild acidification and by phosphate’s ability to form complexes with calcium ions, thereby transiently reducing the concentration of free ionized calcium [31, 32]. As calcium is essential for the activation of multiple coagulation factors and enzyme complexes (e.g., prothrombinase and tenase), phosphate-induced calcium binding might slow thrombin generation. Once sufficient thrombin is generated, fibrin polymerization proceeds normally, resulting in preserved clot firmness.

It should be noted that ROTEM analysis is performed on citrated blood, and in EXTEM testing the first step involves recalcification with the STARTEM reagent (0.2 M CaCl₂) [8]. As previously discussed, phosphate ions tend to form complexes with calcium ions. The higher phosphate concentration resulting from H₂PO₄⁻ addition therefore might increase calcium binding, even though recalcification is carried out to initiate coagulation.

#### Lysis inhibition

Several enzymatic cascades are responsible for the activation of plasminogen into plasmin, the central enzyme in fibrinolysis. This process involves a number of regulatory proteins, including tissue plasminogen activator (tPA), protein C, and protein S. Among these, both plasmin and tPA are particularly sensitive to pH changes, exhibiting optimal enzymatic activity at a physiological pH of approximately 7.5 [33, 34]. The inactivation of tPA is caused by protonation of a histidine molecule in the catalytic center [34]. Furthermore, inhibitory proteins like plasminogen activator inhibitor (PAI) and α2-Antiplasmin are stabilised in acidosis amplifying the inactivation of Plasmin [35, 36]. 

In our ex-vivo experiments, we observed that the maximum lysis (ML) parameter decreased progressively as the pH level dropped. This suggests that acidic conditions impair the fibrinolytic potential of blood by reducing the activity of key enzymes such as plasmin and tPA. As the pH shifts away from their optimal range, the efficiency of fibrin degradation diminishes, leading to more stable clots and reduced lysis.

### Respiratory acidosis model

White et al. assessed patients with respiratory acidosis secondary to acute COPD exacerbations and patients with diabetic keto-acidosis, presenting with a mean pH of 7.08 ± 0.10 at baseline. Despite this pronounced hypercapnia and acidemia, thromboelastographic parameters, including R, K, α-angle, and MA, remained within normal limits and exhibited no significant changes over 24 h, indicating that elevated CO₂ and diabetic keto-acidosis did not impair coagulation dynamics as assessed by TEG [37]. These observations are consistent with our ex-vivo findings. The absence of measurable alterations may reflect the distinct pathophysiology of respiratory versus metabolic acidosis. Furthermore, TEG as well as ROTEM predominantly assesses humoral coagulation and may not capture endothelial or inflammatory contributions relevant in respiratory disease [37]. 

### Limitations of the study

This study offers several notable strengths. It systematically investigated the effects of the acids HCl, C_3_H_6_O_3_, H₃PO₄, and H₂PO₄⁻ at varying concentrations, allowing for a nuanced understanding of acid-specific effects on ROTEM tests independent of pH alone. The use of ROTEM at physiological temperature (37 °C) enhances clinical relevance, and the thorough device validation ensured high measurement precision. The inclusion of CO₂ incubation to model respiratory acidosis adds further clinical significance, distinguishing it from purely metabolic models.

However, as an ex-vivo observational study, certain limitations must be acknowledged. Most notably, the absence of cellular components such as endothelium and inflammatory mediators limits the applicability of findings to in vivo conditions. The relatively small number of participants per subgroup reduces the statistical power and increases variability in some parameters, particularly ML and CFT. Additionally, while functional parameters like MCF and ML were assessed, no direct measurements of fibrinogen, platelet count, or individual coagulation factors were conducted, making it difficult to pinpoint the exact mechanisms underlying observed changes. Finally, the potential for acid-induced haemolysis or protein denaturation, particularly with highly concentrated solutions, and the binding of calcium ions, was not explicitly evaluated, although it may influence ROTEM parameters. A target pH of 7.0 could not be achieved with acid concentrations ≤ 0.01 M, as more than 200 µL of acid would have been required to be added to 800 µL of blood, resulting in significant dilution and unfeasible results. Finally, acidosis impairs platelet function which can only partly be assessed with ROTEM tests [2, 38]. These limitations reflect the inherent constraints of an observational ex vivo design but do not diminish the value of the mechanistic insights gained.

## Conclusion

The results of our study demonstrate that both the type of acid and its concentration significantly influence ROTEM parameters. Hydrochloric acid markedly reduced clot firmness, whereas phosphate-induced acidosis delayed clot initiation and attenuated fibrinolysis under severe acidotic conditions. These acid-specific effects were concentration-dependent and increased with acidosis severity, underscoring the importance of careful selection and characterisation of acidosis models when interpreting viscoelastic coagulation assays. Notably, acidosis induced by CO₂ incubation did not affect clotting time or clot firmness but was associated with reduced fibrinolysis.

## Supplementary Information

Below is the link to the electronic supplementary material.

Supplementary Material 1

## Data Availability

The datasets used and analysed during the current study are available from the corresponding author on reasonable request.
