# Supplementary material for: Impact of acid selection on thrombelastometric test results - an in-vitro cohort study
Source: Thromb J. 2026 Aug 20;24:77. doi: 10.1186/s12959-026-00912-2 (PMC13495198; doi:10.1186/s12959-026-00912-2)
Supplement: Supplementary file 1 [file 12959_2026_912_MOESM1_ESM.docx]

Supplement table 1 Overview of the various volumes of acids required to adjust the respective pH levels

|  |  | **1 M** | | **0.5 M** | | **0.1 M** | |
| --- | --- | --- | --- | --- | --- | --- | --- |
|  | **pH** | **Volume [µL]** | **measured pH** | **Volume [µL]** | **measured pH** | **Volume [µL]** | **measured pH** |
| **HCl** | **7.2** |  |  | 14.9±4.5 | 7.206±0.007 |  |  |
|  | **7.1** |  |  | 21.1±4.8 | 7.103±0.013 |  |  |
|  | **7.0** | 15.1±2.7 | 6.990±0.020 | 29.1±4.9 | 7.000±0.011 | 139.0±14.6 | 7.007±0.005 |
|  | **6.9** |  |  | 34.6±5.1 | 6.905±0.011 |  |  |
| **C3H6O3** | **7.2** |  |  | 19.2±7.3 | 7.202±0.011 |  |  |
|  | **7.1** |  |  | 22.5±4.3 | 7.111±0.004 |  |  |
|  | **7.0** | 16.7±2.7 | 6.989±0.017 | 33.9±4.6 | 7.003±0.012 | 175.0±23.2 | 7.005±0.017 |
|  | **6.9** |  |  | 38.5±6.3 | 6.901±0.010 |  |  |
| **H3PO4** | **7.2** |  |  | 7.2±1.3 | 7.196±0.009 |  |  |
|  | **7.1** |  |  | 9.7±1.9 | 7.104±0.010 |  |  |
|  | **7.0** | 7.6±1.6 | 7.002±0.006 | 16.2±3.2 | 7.001±0.011 | 83.4±15.7 | 7.000±0.010 |
|  | **6.9** |  |  | 18.9±2.3 | 6.902±0.010 |  |  |
| **H2PO4-** | **7.2** | 11.4±2.0 | 7.193±0.012 |  |  |  |  |
|  | **7.1** | 17.0±4.6 | 7.097±0.027 |  |  |  |  |
|  | **7.0** | 26.9±6.4 | 7.003±0.010 | 65.4±7.8 | 7.006±0.006 | 200±0 | 7.131±0.016 |
|  | **6.9** | 36.5±5.9 | 6.914±0.025 |  |  |  |  |

Table footnote: native pH was 7.464±0042

This table lists the various acids investigated and their concentrations, as well as the volumes required to achieve the specified pH values.
